# A Case of Jaundice

**Published:** 1898-06

**Authors:** J. M. Selfridge

**Affiliations:** Oakland, Cal.


### A CASE OF JAUNDICE.

J. M. Selfridge, M. D., Oakland, Cal.

On the evening of November 22d, 1896, I was called to see W. T——, a tall but not fleshy man, aged 69 years.

I was informed that he had been sick six weeks, under the care of Dr. R——, of Oakland, an allopath of good professional standing, who had done everything he knew, and proposed to the patient to do a surgical operation as the only means of relieving the obstruction in the bile-duct, as he told me afterwards, to start the bile, but failed. During these weeks of purgation, etc., he had become very weak, and when I saw him he was in bed, without strength to sit up. The conjunctivæ were yellow, and the skin a light colored bronze, his urine the color of black coffee, and his stools the color of clay. They were long and slender, like pipe-stems, and were evacuated with much straining. The liver was enlarged, and there was some tenderness on pressure. There was loss of appetite, and when he took anything into his stomach he could retain it but about half an hour, when he was obliged to vomit it. He was cheerful, but his mind was sluggish and his sleep disturbed by horrible dreams.

Treatment.—The inability to keep anything on his stomach longer than half an hour was the keynote to the remedy indicated. Then, again, another very unusual and peculiar symptom was the stool—long and slender like pipe-stems, and voided with much straining. Now, so far as I know, there is but *one* remedy in the materia medica that has the peculiar vomiting already mentioned, accompanied by such a contraction of the internal sphincter that stools are forced through like pipe-stems, with great straining. Graphites has "very thin stools like round worms," Alumina has stools like pipe-stems, but they do not have the characteristic vomiting and icteroid symptoms like Phophorus, which in this case was the remedy, and it was administered in the 200th potency in water, a teaspoonful every two hours, except when asleep.

November 23d I found my patient about the same, except that he did not vomit so frequently. Daylight, however, revealed another symptom. The appearance of his gums satisfied me that he had been taking massive doses of some form of Mercury, and I so informed him. He thought not—"Doctors did not salivate people nowadays." He said the doctor had been giving him some small tablets for about a week, but he did not know what they were. To satisfy myself I telephoned the doctor that evening, and asked him what those tablets contained that he had been giving Mr. T——. He answered very promptly Calomel.

November 24th I found my patient entirely relieved of the vomiting, but there was no doubt of the fact that he was salivated. His gums were not only red, swollen, and spongy, with fetor of the breath, but the saliva was flowing freely—he had saturated six handkerchiefs during the night. Now, although Phos. has much watery saliva in the mouth, saliva increased, etc.; still, on account of this medicinal disease, I felt obliged to omit the otherwise indicated remedy and give a remedy for the Mercury. After a little thought I gave Nitric-acid 6x in water, every three hours a teaspoonful, except when asleep. This remedy had to be continued sixteen days before the symptoms of salivation had disappeared sufficiently to resume the remedy indicated for the jaundiced condition. December 13th he still had the pipe-stem stools, voided with much straining, which, with the other symptoms, indicated Phos. as the remedy, and it was again administered every three hours. For food he could take only malted milk and meat juice. In a week the urine began to grow lighter colored and the stools to show the presence of bile, and as the sphincter became relaxed the form of the stool changed, and there was less straining. By the 28th every symptom of his former illness had disappeared, except the color of the skin—the pigment was still tinged with bile.

